# Long Non-coding RNA *LINC01969* Promotes Ovarian Cancer by Regulating the miR-144-5p/LARP1 Axis as a Competing Endogenous RNA

**DOI:** 10.3389/fcell.2020.625730

**Published:** 2021-02-04

**Authors:** Jinxin Chen, Xiaocen Li, Lu Yang, Jingru Zhang

**Affiliations:** ^1^Department of Gynecology, Liaoning Cancer Hospital and Institute, Cancer Hospital of China Medical University, Shenyang, China; ^2^Department of Graduate School, Dalian Medical University, Dalian, China; ^3^Medical Oncology Department of Gastrointestinal Cancer, Liaoning Cancer Hospital and Institute, Cancer Hospital of China Medical University, Shenyang, China

**Keywords:** lncRNA, ovarian cancer, epithelial-mesenchymal transition, cell proliferation, invasion

## Abstract

Accumulating evidence has shown that long non-coding RNAs (lncRNAs) can be used as biological markers and treatment targets in cancer and play various roles in cancer-related biological processes. However, the lncRNA expression profiles and their roles and action mechanisms in ovarian cancer (OC) are largely unknown. Here, we assessed the lncRNA expression profiles in OC tissues from The Cancer Genome Atlas (TCGA) database, and one upregulated lncRNA, *LINC01969*, was selected for further study. *LINC01969* expression levels in 41 patients were verified using quantitative real-time polymerase chain reaction (qRT-PCR). The *in vitro* effects of *LINC01969* on OC cell migration, invasion, and proliferation were determined by the CCK-8, ethynyl-2-deoxyuridine (EdU), wound healing, and Transwell assays. Epithelial–mesenchymal transition (EMT) was evaluated using qRT-PCR and Western blotting. The molecular mechanisms of *LINC01969* in OC were assessed through bioinformatics analysis, RNA-binding protein immunoprecipitation (RIP), dual luciferase reporter gene assays, and a rescue experiment. Finally, *in vivo* experiments were conducted to evaluate the functions of *LINC01969*. The results of the current study showed that *LINC01969* was dramatically upregulated in OC, and patients with lower *LINC01969* expression levels tended to have better overall survival. Further experiments demonstrated that *LINC01969* promoted the migration, invasion, and proliferation of OC cells *in vitro* and sped up tumor growth *in vivo*. Additionally, *LINC01969*, which primarily exists in the cytoplasm, boosted *LARP1* expression by sponging miR-144-5p and promoted the malignant phenotypes of OC cells. In conclusion, the *LINC01969*/miR-144-5p/LARP1 axis is a newly identified regulatory signaling pathway involved in OC progression.

## Introduction

Ovarian cancer (OC) ranks second among lethal gynecological cancers worldwide (Lheureux et al., 2019). According to estimates, 238,719 women worldwide were newly diagnosed with OC in 2012, and 151,917 died from OC (Iversen et al., 2018). Despite progress in surgical treatment and chemoradiotherapy, the survival rate of OC patients has only changed modestly, and the 5-year survival rate is only 47%, even in the USA, Canada, and other wealthy countries (Burki, 2017; Torre et al., 2018). As the anatomical characteristics of the ovary complicate diagnosis, the disease is often diagnosed in late stage when the survival rate is low (Dafni et al., 2021). Hence, much attention has been paid to the molecular biological mechanisms underlying the occurrence and progression of OC.

Long non-coding RNAs (lncRNAs) are ncRNAs more than 200 nt in length that were recently discovered (Li et al., 2017; Xie et al., 2018; Liu et al., 2019b), and their functions are just now being explored. It has been reported that lncRNAs play important roles in multiple biological and pathogenic processes, such as genomic imprinting (Liu et al., 2017; Sanli et al., 2018), cancer metastasis (Kim et al., 2018; Zhuo et al., 2019; Qiu et al., 2020), stem cell differentiation (Daneshvar et al., 2016; Hou et al., 2017; Zhang et al., 2019), and X chromosome inactivation (Tian et al., 2010; Furlan and Rougeulle, 2016; Carter et al., 2020) among many others. LncRNAs function *via* diverse mechanisms, including chromatin modification and cell signaling (Satpathy and Chang, 2015). One of the major functions of lncRNAs is as competing endogenous RNAs (ceRNAs), which sequester microRNAs (miRNAs) from their messenger RNA (mRNA) targets, thereby increasing target gene expression (Ransohoff et al., 2018). For example, in lung cancer, lncRNA *LCAT1* functions as a ceRNA by sponging miR-4715-5p to control RAC1 function (Yang et al., 2019). LncRNA MNX1-AS1 induces lung cancer progression through the miR-527/BRF2 pathway (Liu et al., 2019a). LncRNA *AK002107* inversely controls miR-140-5p and triggers EMT in OC by targeting *TGFBR1* (Tang et al., 2019). LncRNA *AC010789.1* promotes colorectal cancer progression by targeting the microRNA-432-3p/ZEB1 axis and the Wnt/β-catenin signaling pathway (Duan et al., 2020). A study of the BRCA1/2 ceRNA network in OC patients with wild-type BRCA1/2 revealed a novel three-lncRNA signature that could predict both prognosis and chemo-response (Zhang et al., 2020).

An assessment of the differentially expressed lncRNAs in OC using The Cancer Genome Atlas (TCGA) database showed that *LINC01969* is expressed at high levels in OC and is associated with patient survival. However, whether *LINC01969* correlates with the onset and development of OC is unknown. In the current study, *LINC01969* expression levels in OC and non-cancer tissues were compared, which showed that *LINC01969* expression was markedly higher in OC. Functional studies showed that *LINC01969* acts as an oncogene to promote OC through the miR-144-5p/LARP1 axis.

## Materials and Methods

### Patients and Clinical Sampling

In 2016–2018, 41 paired fresh tumor and non-tumor tissues were harvested from patients at Liaoning Cancer Hospital and Institute and snap frozen at −80°C (Table 1). Written informed consent was obtained from all patients, and the study was approved by the Ethnics Committees of Liaoning Cancer Hospital and Institute.

**Table 1:** Association of *LINC01969* expression with clinicopathological features of ovarian cancer.

**Feathers**	**Number**	**High**	**Low**	***P*-value**
All cases	41	19	22	
Age (years)				0.7579
<50	20	10	10	
≥50	21	9	12	
Tumor size (cm)				**0.0252**
<4	17	4	13	
≥4	24	15	9	
Lymph node metastasis				**0.0214**
Negative	15	3	12	
Positive	26	16	10	
FIGO stage				**0.0127**
I/II	22	6	16	
III/IV	19	13	6	

*Total data from 41 tumor tissues of ovarian cancer patients were analyzed. For the expression of LINC01969 was assayed by qRT-PCR, the average expression level was used as the cutoff. Data were analyzed by chi-squared test and Fisher's exact test. P-value in bold indicates statistically significant*.

### Cell Culture

Human OC cell lines (A2780, OVCAR-3, SKOV3, and TOV112D) and a normal human ovarian cell line (IOSE-80) (ATCC, Manassas, VA, USA) were cultured in Dulbecco's modified Eagle's medium (DMEM) (HyClone, Beijing, China) containing 10% fetal bovine serum (FBS) (HyClone).

### Cell Transfection

A *LINC01969* short hairpin RNA (shRNA) (sh-*LINC01969*) and negative control shRNA (sh-NC) were designed, synthesized, and inserted into plasmid vector PLKO.1-puro (BioVector NTCC Inc., Beijing, China). To overexpress *LINC01969*, the *LINC01969* genomic sequence was amplified and inserted into pLV-CMV-EF1a-GFP-T2A-Puro vector. *LARP1* small-interfering (si-*LARP1*), negative control (si-NC), and miRNA mimics and inhibitors were provided by RiboBio (Guangzhou, China). After 24 h of culture, the cells underwent a 48-h transient transfection with the corresponding vector using Lipofectamine 3000 transfection reagent (Invitrogen, Shanghai, China) according to the manufacturer's instructions. The transfected cells were analyzed by quantitative real-time PCR (qRT-PCR). Assays were carried out at least thrice.

### RNA Sequestering and qRT-PCR

Total RNA was extracted from cells using TRIzol reagent (Invitrogen, Shanghai, China) and was reverse transcribed using the PrimerScript RT-PCR kit (Takara, Dalian, China) according to the manufacturer's instructions. Then, RNA levels were assessed by qRT-PCR using the TaqMan MiRNA Assay Kit (Applied Biosystems). The relative expression of targets was determined in triplicate on an ABI 7500 RT-PCR system (Applied Biosystems). β-Actin or U6 small nuclear RNA (snRNA) was used as a reference gene for normalization of miRNA or mRNA expression. The delta Ct method was used to calculate the relative expression. The primers used in this study are shown in Supplementary Table 1.

### Chromatin Fractionation

Cytoplasmic and nuclear RNA were extracted with the PARIS Kit (Life Technologies, USA), and cytoplasm and nuclear RNA levels were assessed by qRT-RCR using glyceraldehyde 3-phosphate dehydrogenase (GAPDH) and U6 as internal references, respectively.

### Cell Counting Kit-8 Assay

The Cell Counting Kit-8 from Dojindo (Beijing, China) was utilized to assess cell proliferation according to the manufacturer's instructions. The cells were seeded in 96-well plates (2 × 10^3^ cells/well) in 100 μl of medium in quintuplicate. After 1, 2, 3, and 4 days, Cell Counting Kit-8 (CCK8) reagent was added to the wells and incubated for 2.5 h. Then, the absorbance was measured at 450 nm.

### Ethynyl-2-Deoxyuridine Assay

Cell proliferation was evaluated using the ethynyl-2-deoxyuridine (EdU) Apollo DNA *in vitro* kit (RiboBio, Guangzhou, China) according to the manufacturer's instructions. OC cells (1 × 10^4^) were seeded in each well of 96-well plates for transfection. After incubation at 37°C and 5% CO_2_ for 48 h, cells were added with 20 μM EdU and incubated for another 2 h. OC cells were fixed with 4% paraformaldehyde and dyed with Apollo 567 and Hoechst 33342 (Hu et al., 2017). The number of cells was counted using Image J software (NIH, Bethesda, MD, USA). The cell proliferation rate was calculated according to the manufacturer's instructions.

### Wound Healing Assay

Wounds were generated with a 200-μl pipette tip in a cell monolayer at 80% confluence. After washing with phosphate-buffered saline (PBS), medium containing 20 μg/ml mitomycin C without FBS was added to assess the impact of various genes on cell proliferation. At 0 and 24 h after wounding, pictures were taken to assess healing.

### Transwell Assay

The invasion ability of OC cells was probed using a Transwell apparatus. Briefly, OC cells in DMEM containing 1% FBS were seeded at 10^5^ cells/well in the upper chamber of a Transwell apparatus (8-μm pore size; Corning, Corning, NY, USA) precoated with Matrigel (1:6 dilution; Corning). DMEM containing 10% FBS was added to the lower chamber. After a 24-h incubation at 37°C, the migrated or invaded cells in the lower chamber were fixed with 4% methanol and stained with crystal violet. The cells in five random fields of a microscope (100×) were counted. All assays were performed thrice.

### Western Blot Assay

Total cell lysates were prepared in a 1 × sodium dodecyl sulfate buffer, and total proteins were separated by sodium dodecyl sulfate–polyacrylamide gel electrophoresis and transferred onto nitrocellulose membranes. The membranes were blocked with skim milk (5%) and incubated with primary antibody at 4°C overnight. The primary antibodies used were against β-actin (1:1,000, ab8226), LARP1 (1:1,000, ab86359), E-cadherin (1:50, ab1416), Snail (1:1000, ab216347), and Vimentin (1:1,000, ab8069) (Abcam, Shanghai, China). The membranes were then incubated with antirabbit or antimouse secondary antibodies (1:1,000, Abcam, Shanghai, China) and visualized by ECL. All assays were performed at least thrice.

### RNA Fluorescence *in situ* Hybridization

A fluorescence *in situ* hybridization (FISH) assay was performed using the Ribo™ FISH Kit (RiboBio, China) according to the manufacturer's instructions. The Cy3-conjugated *LINC01969* probe was designed and synthesized by RiboBio. Fluorescence was detected with a confocal laser-scanning microscope (Leica, Germany).

### RNA-Binding Protein Immunoprecipitation Assay

An RNA-binding protein immunoprecipitation (RIP) assay was performed using the EZ-magna RIP kit (Millipore, China) according to the manufacturer's instructions. First, A2780 and SKOV3 cells were harvested and lysed in complete RIP lysis buffer. Then, the cell extracts were incubated with RIP buffer containing anti-Argonaute-2 antibody-conjugated magnetic beads (ab32381, Abcam, Shanghai, China); an anti-immunoglobulin G (anti-IgG) antibody (ab6702, Abcam) was used as a control. Then, the samples were incubated with protease K and oscillated to digest proteins and isolate immunoprecipitated RNAs. The RNA concentration was determined by using a NanoDrop spectrophotometer, and the purified RNAs were evaluated by RT-PCR.

### Dual Luciferase Reporter Gene Assay

*LINC01969* and *LARP1* fragments containing the miR-144-5p binding sites were amplified by PCR and cloned into the pmirGLO vector downstream of the luciferase reporter gene to generate *LINC01969* Wt and *LARP1* Wt. Then, the QuikChange XL Site-Directed Mutagenesis Kit (Stratagene) was utilized to generate *LINC01969* Mut and *LARP1* Mut (with mutations in the miR-144-5p binding sites) according to the manufacturer's guidelines. HEK293T cells were cotransfected with either miR-NC or miR-144-5p mimic and either *LINC01969* Wt/Mut or *LARP1* Wt/Mut. After 48 h of transfection, cells were collected, and a luciferase assay was conducted using the Dual-Luciferase Reporter Assay System (Promega, Beijing, China).

### Implantation of Tumor Xenografts in Nude Mice

Four-week-old female athymic BALB/c nude mice were randomly divided into two groups (*n* = 3/group) and cultured under aseptic conditions with sterile food and water. A xenograft model of OC was generated by subcutaneously injecting A2780 cells into the mice. Tumor volume was measured for 4 weeks and was calculated according to the following formula: volume = (length) × (width)^2^/2. After 4 weeks, the mice were killed, and tumor weight was measured. All animal experiments were approved by the Liaoning Cancer Hospital and Institute and were conducted in accordance with the Guide for the Care and Use of Laboratory Animals (NIH publication 80-23).

### Statistical Analysis

Statistical analyses were conducted using SPSS 16.0 (SPSS, Beijing, China) and GraphPad Prism 5.0 (GraphPad, La Jolla, CA, USA). Data were collected from at least three separate assays and are expressed as means ± SEM. Intergroup differences were evaluated using Student's *t*-test. Normally distributed data were assessed using one-way ANOVA, and non-normally distributed data were assessed using the Mann–Whitney U test. A *P*-value < 0.05 was considered statistically significant.

## Results

### Upregulated *LINC01969* Expression Is Correlated With Unsatisfactory Prognosis in OC

**Figure 1:**
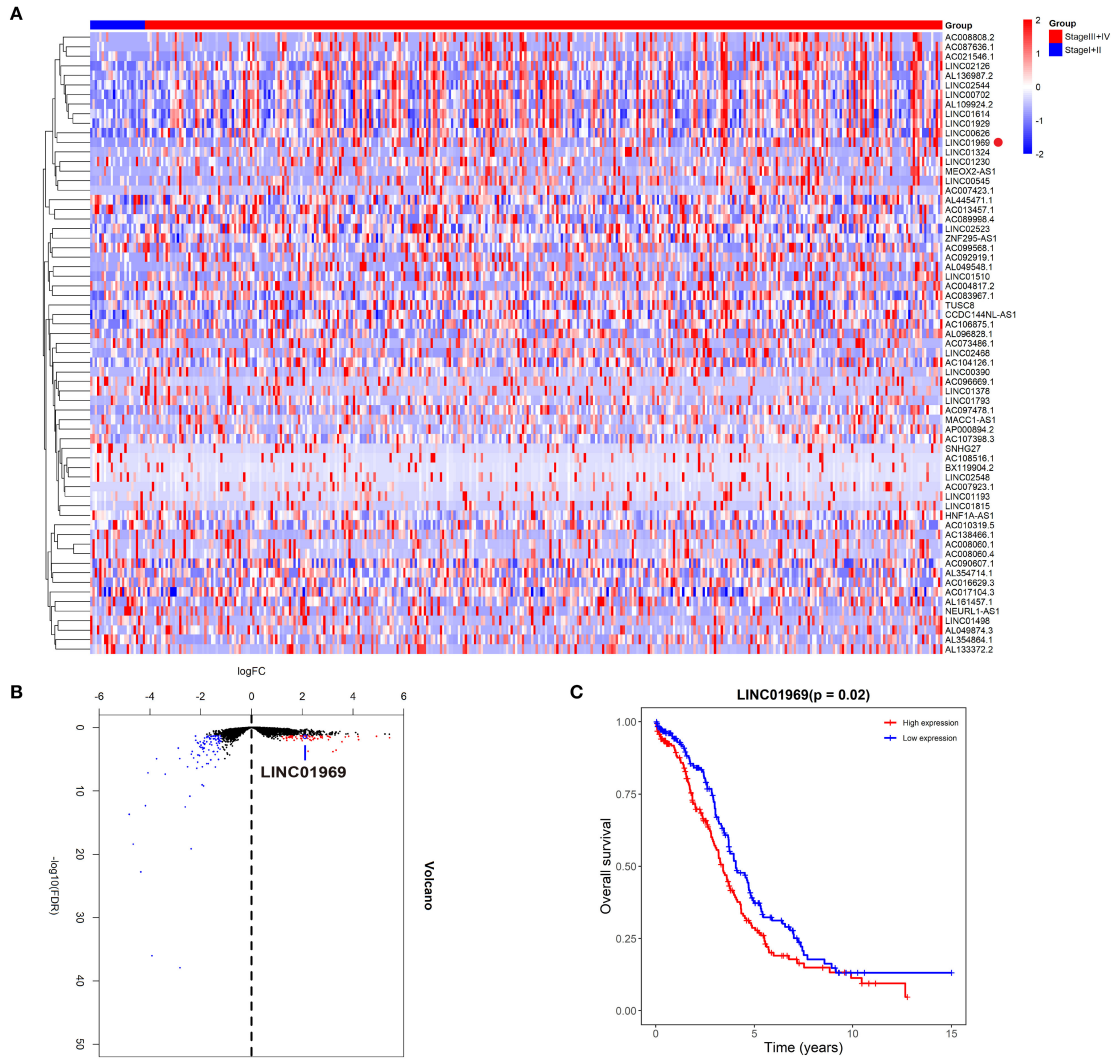
Upregulated *LINC01969* correlates with unsatisfactory prognosis of ovarian cancer (OC) cases revealed by The Cancer Genome Atlas (TCGA) database. As per the Neoplasm American Joint Committee on Cancer Clinical Group Stage, we allocated OC tissues into stage I + II group and stage III + IV group. **(A,B)** The heat and volcano maps revealed all differentially expressed long non-coding RNAs (lncRNAs) of OC tissues in the TCGA database with log2FC >2 or < −2, *P* < 0.05. **(C)** Kaplan–Meier survival analysis of *LINC01969* expression in OC tissues from TCGA database.

We first assessed the lncRNA expression levels in OC tissues in TCGA database. We divided the OC tissues into two groups: a stage I + II group and a stage III + IV group. The heat and volcano maps revealed all differentially expressed lncRNAs with a log2FC >2 or < −2, *P* < 0.05 (Figures 1A,B). We chose the top 15 most highly expressed lncRNAs in stage III + IV compared to stage I + II and analyzed their impact on survival in TCGA. We found that only *LINC01969* was associated with survival in OC, and higher *LINC01969* expression was associated with lower overall survival of OC patients (Figure 1C). These findings indicate that *LINC01969* is probably involved in the progression of OC.

### *LINC01969* Expression Is Upregulated in OC Tissues and Cell Lines

**Figure 2:**
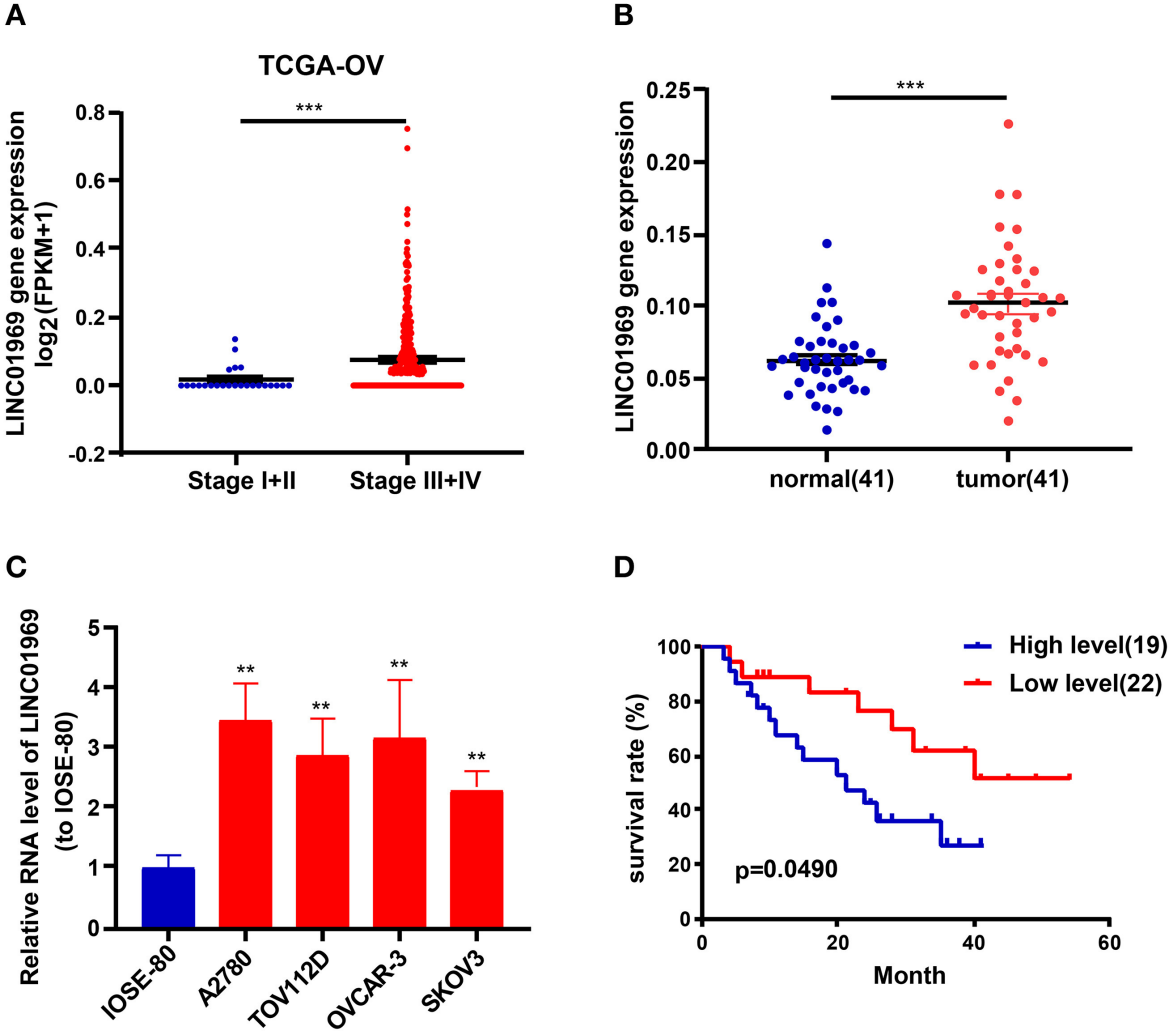
*LINC01969* expression is upregulated in ovarian cancer (OC) tissues and cell lines. **(A)** *LINC01969* expression in OC stage I + II and stage III + IV from TCGA database. **(B)** Quantitative real-time PCR (qRT-PCR) results indicated that *LINC01969* was upregulated in OC tissues (*n* = 41) compared with paired non-normal tissues (*n* = 41). **(C)** *LINC01969* expression in OC cell lines and IOSE-80 cell line was analyzed by qRT-PCR. **(D)** Kaplan–Meier curves of overall survival (OS) in 41 OC patients based on *LINC01969* level (average expression value was the cutoff). Data represent the mean ± SD; **P < 0.01, ***P < 0.001.

Analysis of TCGA data revealed that *LINC01969* was expressed at a dramatically lower level in OC stage I + II tumors than in OC stage III + IV tumors (Figure 2A). qRT-PCR analysis of 41 OC samples revealed significantly higher *LINC01969* levels in OC tissues than in paired non-normal tissues (Figure 2B). *LINC01969* expression was also assessed in OC cell lines, which showed that *LINC01969* was expressed at markedly higher levels in OC cell lines (A2780, TOV112D, OVCAR-3, and SKOV3) than in normal human cells (IOSE-80) (Figure 2C). The role of *LINC01969* as a biomarker for prognosis was determined by dividing the 41 OC tissues into high and low *LINC01969* expression groups based on the average *LINC01969* expression. Kaplan–Meier analysis revealed that *LINC01969* overexpression was related to lower overall survival (Figure 2D). These findings imply that *LINC01969* might be a useful biomarker for OC prognosis.

### *LINC01969* Promotes OC Cell Growth

**Figure 3:**
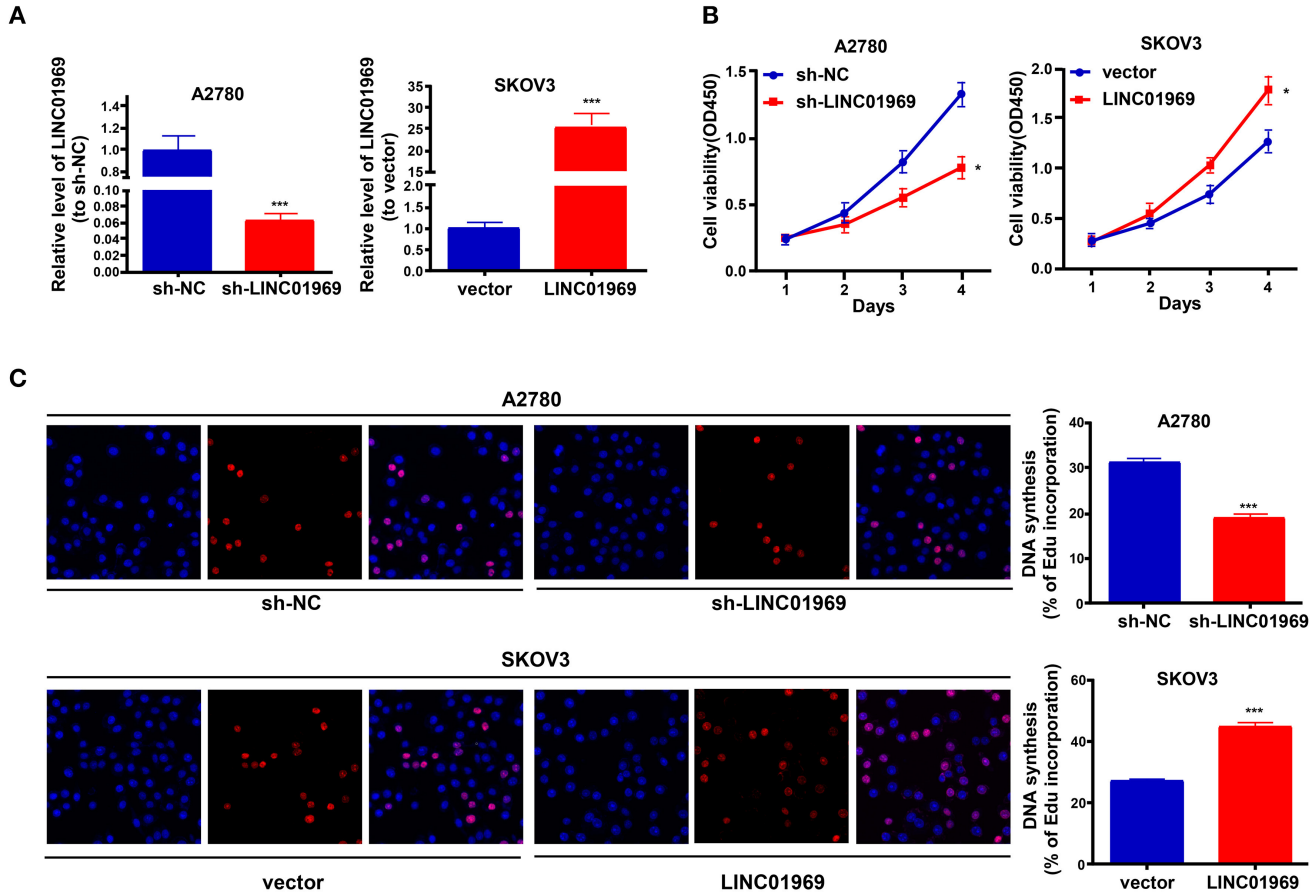
*LINC01969* promotes cell growth in ovarian cancer (OC) cells. **(A)** Transfection efficiency of sh-*LINC01969* and *LINC01969*-overexpressing vector. **(B)** Cell viability was analyzed by CCK-8 assay. **(C)** EdU assay indicated that *LINC01969* silencing delayed OC cell proliferation. Data were presented as represent the mean ± SD of three independent experiments; *P < 0.05, ***P < 0.001.

*LINC01969* was expressed at higher levels in OC tissues and was related to unsatisfactory prognosis of OC patients. These findings indicate that *LINC01969* may promote the occurrence and development of OC. To verify this, *LINC01969* expression in A2780 OC cells was knocked down with a shRNA (Figure 3A), and *LINC01969* was overexpressed in SKOV3 OC cells with a *LINC01969*-overexpressing lentivirus (Figure 3A). Then, the CCK8 and EdU assays were implemented to assess the proliferation of A2780 and SKOV3 cells. The CCK-8 assay showed that *LINC01969* knockdown significantly suppressed proliferation in A2780 cells, while overexpression of *LINC01969* markedly promoted proliferation in SKOV3 cells (Figure 3B). The results of the EdU assay confirmed the impact of *LINC01969* on OC proliferation (Figure 3C).

### *LINC01969* Promotes Migration, Invasion, and EMT in OC Cells

**Figure 4:**
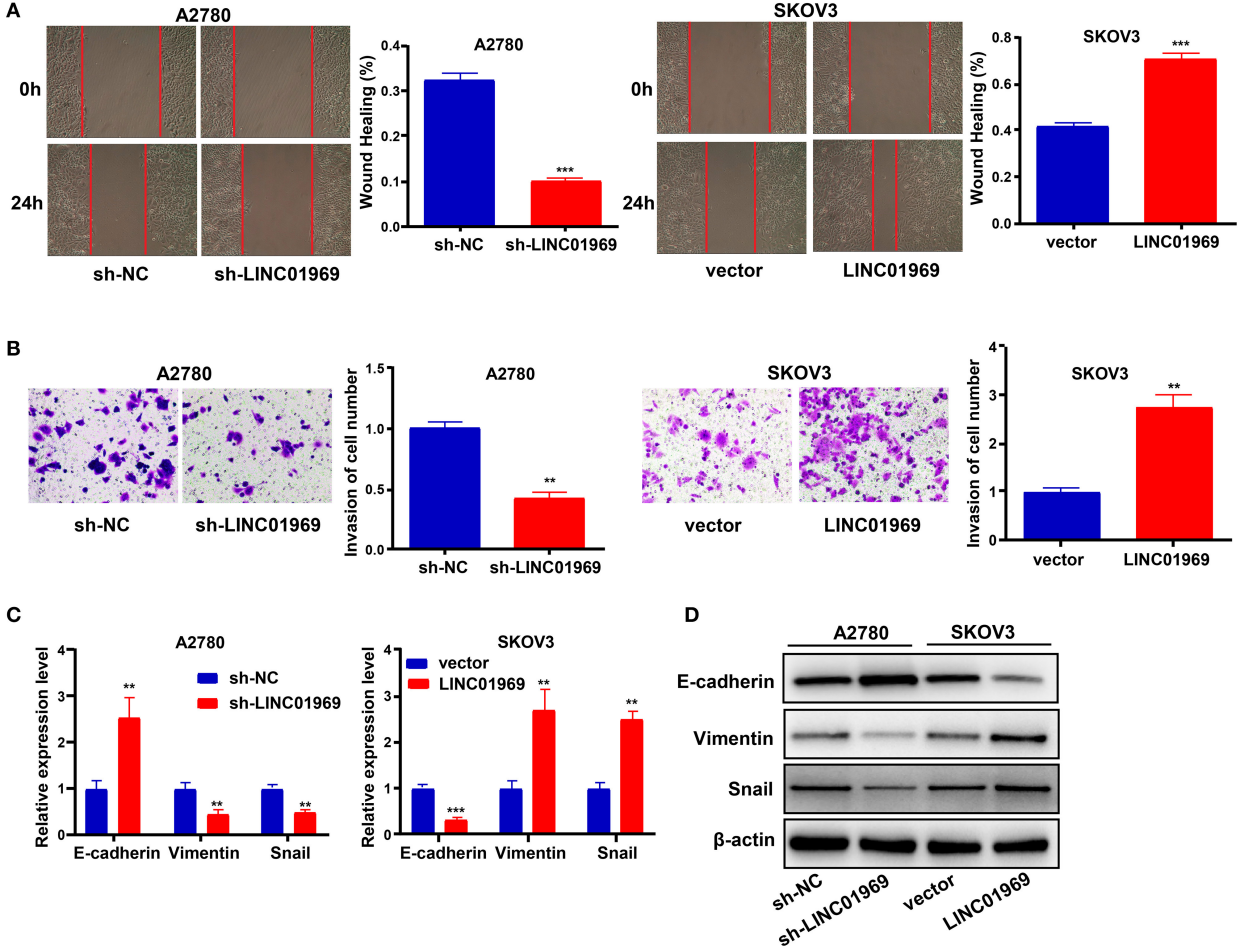
*LINC01969* promotes the cell migration, invasion, and epithelial–mesenchymal transition (EMT) of ovarian cancer (OC) cells. **(A)** Wound healing assay showed that *LINC01969* silencing suppressed the ability of OC cell migration. **(B)** Transwell assay showed that *LINC01969* silencing suppressed the ability of OC cell invasion. **(C,D)** The expression of EMT markers were determined using quantitative real-time PCR (qRT-PCR) and Western blot assay. Data were presented as mean ± SD of three independent experiments; **P < 0.01, ***P < 0.001.

We used wound healing and Transwell assays to test whether *LINC01969* modulated the migration and invasion ability of OC cells. *LINC01969*-overexpressing SKOV3 cells migrated at a higher rate than the control cells, whereas *LINC01969* knockdown A2780 cells barely migrated (Figure 4A). As revealed by the Transwell assay results, cells that express high levels of *LINC01969* had stronger invasion capability (Figure 4B). Next, we evaluated the effect of *LINC01969* on OC cell EMT by measuring EMT marker expression using qRT-PCR and Western blotting. The results showed that knockdown of *LINC01969* increased E-cadherin expression but decreased Snail and Vimentin expression levels in OC cells (Figures 4C,D). These results indicate that *LINC01969* probably affects OC cell invasion and migration.

### *LINC01969* Is Targeted by miR-144-5p

**Figure 5:**
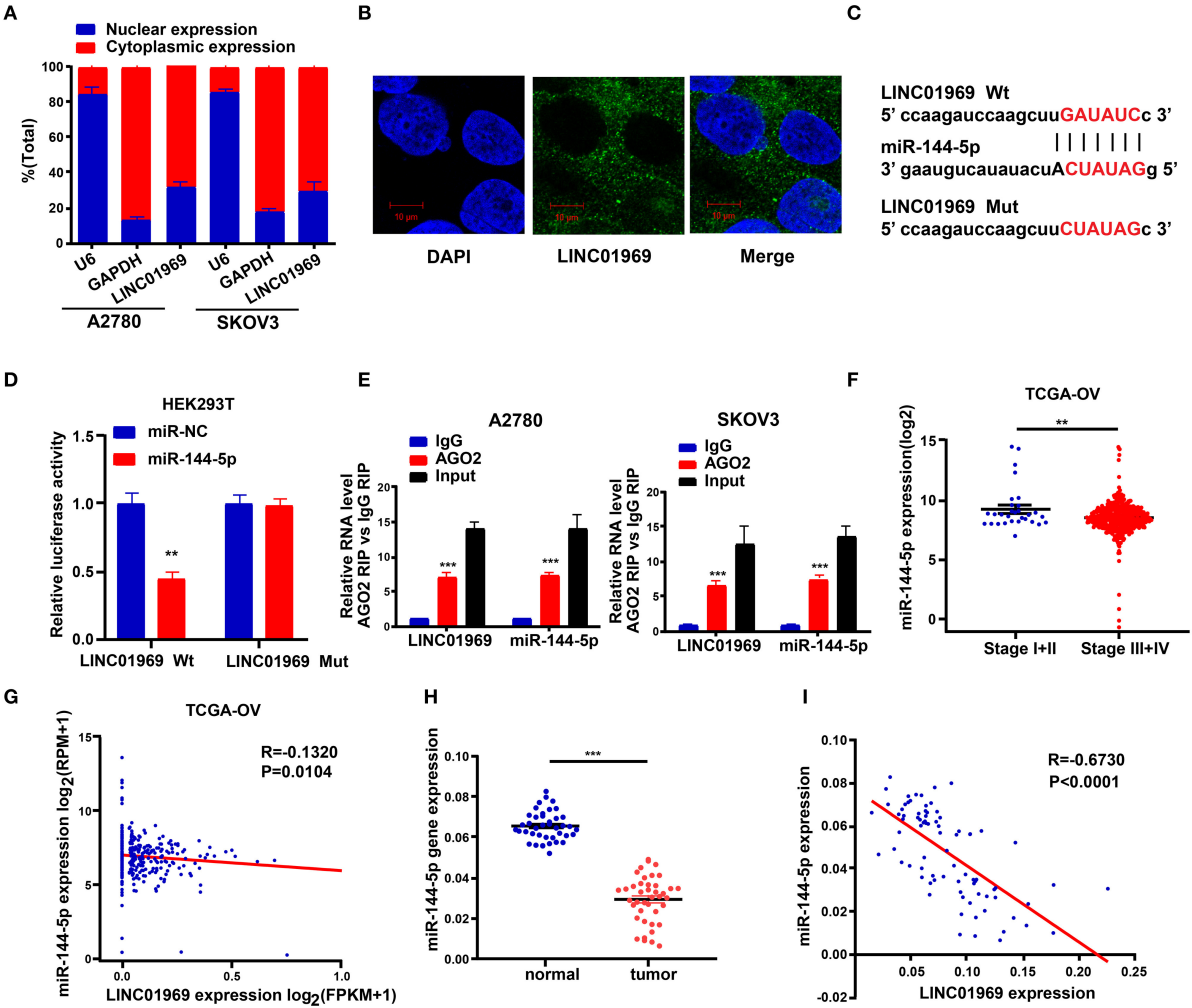
*LINC01969* is targeted by miR-144-5p. **(A)** Localization of *LINC01969* by nucleocytoplasmic separation experiment. **(B)** RNA-FISH in A2780 cells. **(C)** Putative miR-144-5p binding sequence and mutation sequence of *LINC01969* messenger RNA (mRNA) were as shown. **(D)** Dual luciferase reporter assay was used to confirm the direct target between *LINC01969* and miR-144-5p. **(E)** RNA-binding protein immunoprecipitation (RIP) assay was used to detect whether miR-144-5p could bind with *LINC01969*. **(F)** MiR-144-5p expression in ovarian cancer (OC) stage I + II and stage III + IV, from The Cancer Genome Atlas (TCGA) database. **(G)** The correlation analysis between miR-144-5p expression and *LINC01969* expression in OC from TCGA database. **(H)** MiR-144-5p expression in OC tissues and paired non-normal tissues. **(I)** The correlation analysis between miR-144-5p expression and *LINC01969* expression in 41 OC tissues and 41 adjacent no normal tissues. Data were presented as mean ± SD; **P < 0.01, ***P < 0.001.

The subcellular location of an lncRNA is intimately correlated with its biological and molecular functions (Wen et al., 2018). Hence, nucleo-cytoplasmic sequestering and RNA-FISH assays were used to determine the subcellular distribution of *LINC01969*. The results showed that most of the *LINC01969* signal is located within the cytoplasm, but some signal was detected in the nucleus (Figures 5A,B). Then, we looked for possible *LINC01969* targets to determine its functional mechanism using starBase (http://starbase.sysu.edu.cn), which identified miR-144-5p as a potential target. The miR-144-5p binding sites in *LINC01969*-Wt and *LINC01969*-Mut are shown in Figure 5C. According to the starBase results, the binding site is located at chr17:48292600-48292621 within the lncRNA. The binding region is exon-3.

A dual luciferase reporter assay showed that cotransfection of the *LINC01969*-Wt luciferase reporter construct with miR-144-5p mimics significantly reduced luciferase activity, while cotransfection of the *LINC01969*-Mut luciferase reporter construct with miR-144-5p mimics did not affect luciferase activity in HEK293T cells (Figure 5D). The interaction of miR-144-5p and *LINC01969* was confirmed *via* RIP assay, which showed that, relative to those in IgG, *LINC01969* and miR-144-5p were enriched in the AGO2-containing miRNA ribonucleoprotein complexes (Figure 5E). TCGA database analysis showed that miR-144-5p expression was higher in stage I + II OC than in stage III + IV OC (Figure 5F), and *LINC01969* expression levels were inversely correlated with miR-144-5p expression in OC samples (Figure 5G). Consistently, miR-144-5p gene expression levels in the 41 OC samples in our dataset were higher than in the non-cancer samples (Figure 5H), and *LINC01969* expression was inversely related to miR-144-5p expression in the OC samples (Figure 5I). Based on these data, we concluded that *LINC01969* is targeted by miR-144-5p.

### *LINC01969* Controls *LARP1* Expression by Acting as a ceRNA for miR-144-5p to Promote OC

**Figure 6:**
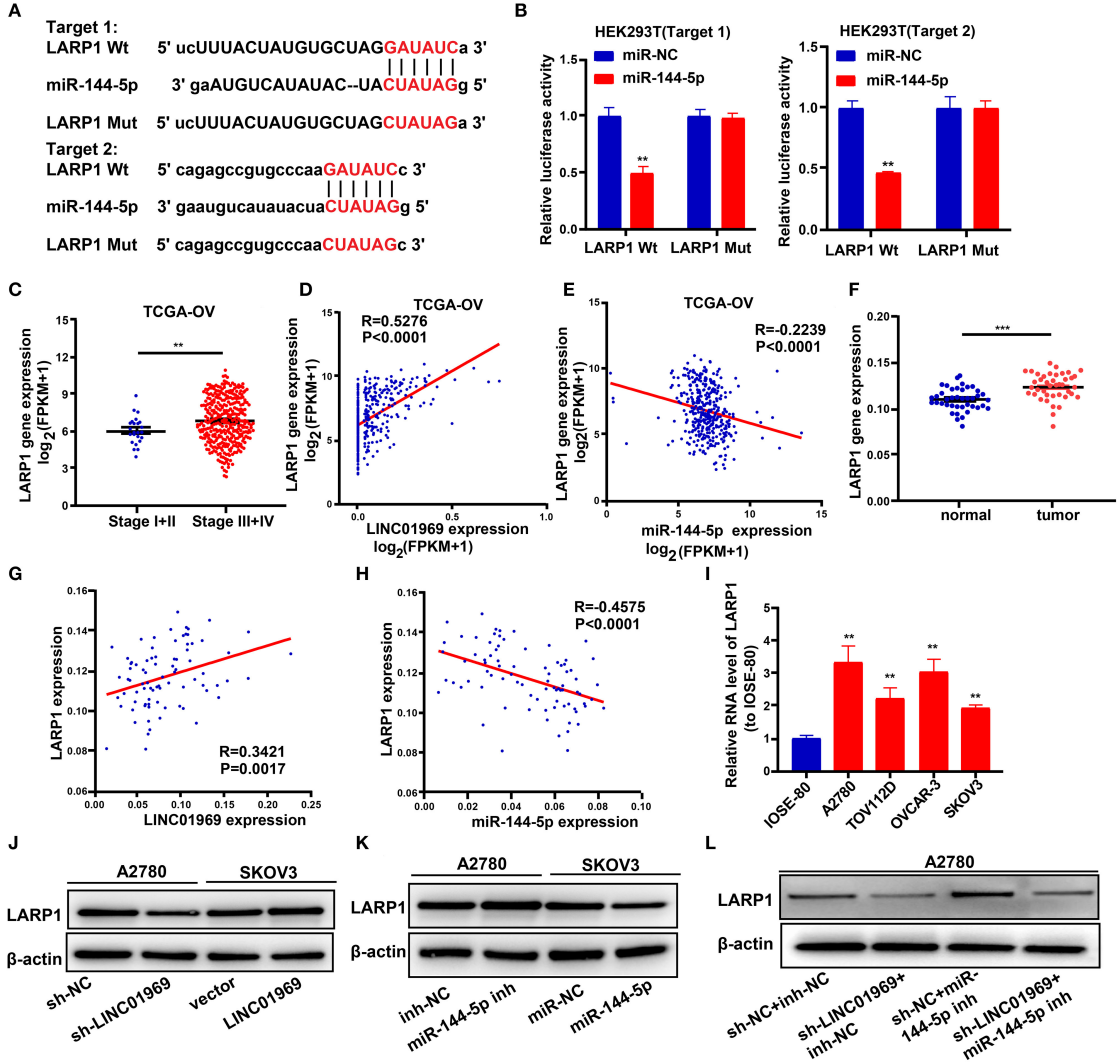
*LINC01969* regulates LARP1 by acting as a competing endogenous RNA (ceRNA) for miR-144-5p. **(A)** Two potential miR-144-5p binding sequences and mutation sequences of *LARP1* messenger RNA (mRNA) were shown. **(B)** Dual luciferase reporter assays were used to confirm the direct target between *LARP1* and miR-144-5p. **(C)** *LARP1* expression in ovarian cancer (OC) stage I + II and stage III + IV from The Cancer Genome Atlas (TCGA) database. **(D)** The correlation analysis between *LINC01969* expression and *LARP1* expression in OC from TCGA database. **(E)** The correlation analysis between miR-144-5p expression and *LARP1* expression in OC from TCGA database. **(F)** *LARP1* gene expression in OC tissues and paired non-normal tissues from our dataset. **(G)** The correlation analysis between *LINC01969* expression and *LARP1* expression in OC tissues and paired non-normal tissues from our dataset. **(H)** The correlation analysis between miR-144-5p expression and *LARP1* expression in OC tissues and paired non-normal tissues from our dataset. **(I)** *LARP1* expression in OC cell lines and IOSE-80 cell line was analyzed by quantitative real-time PCR (qRT-PCR). **(J–L)** The level of *LARP1* was detected by Western blot assay. Data were presented as mean ± SD; **P < 0.01, ***P < 0.001.

To explore the possible roles of miR-144-5p in OC progression, the target genes of miR-144-5p were screened using starBase, and we found that miR-144-5p may interact with *LARP1*. We then mutated the two potential miR-144-5p binding sites in *LARP1* (Figure 6A), which, according to the starBase search results, are located within the 3′-untranslated region (UTR) of the *LARP1* mRNA. Dual luciferase reporter assays revealed that cotransfection of the *LARP1*-Wt luciferase reporter construct with miR-144-5p mimics reduced luciferase activity, whereas cotransfection of the *LARP1*-Mut luciferase reporter construct with miR-144-5p mimics had no impact on luciferase activity in HEK293T cells (Figure 6B). TCGA database analysis showed that *LARP1* expression was lower in stage I + II OC than in stage III + IV OC (Figure 6C). Our analysis also showed that *LARP1* expression was higher in 41 OC samples than in the normal tissue samples (Figure 6F). Moreover, *LARP1* expression was positively related to *LINC01969* expression (Figures 6D,G) and negatively related to miR-144-5p expression in the OC samples (Figures 6E,H) in both TCGA database and our dataset. We also analyzed its expression in OC cell lines, and the results indicated that *LARP1* expression is significantly higher in the tested OC cell lines (A2780, TOV112D, OVCAR-3, and SKOV3) than in the tested normal human cell line (IOSE-80) (Figure 6I). These data indicate that *LARP1* is a target gene of miR-144-5p.

Next, we used a Western blot assay to explore whether *LINC01969* can regulate *LARP1* expression in OC cells *via* miR-144-5p. The results showed that *LARP1* expression was reduced by *LINC01969* knockdown (sh-*LINC01969*) and miR-144-5p mimics (Figures 6J,K). In contrast, *LARP1* expression was elevated by *LINC01969* overexpression and a miR-144-5p inhibitor (Figures 6I,J). Additionally, *LINC01969* silencing reduced *LARP1* expression in A2780 cells, and this was reversed by knockdown of miR-144-5p (Figure 6L). These findings imply that *LINC01969* controls *LARP1* expression in OC cells in a miR-144-5p-dependent manner.

**Figure 7:**
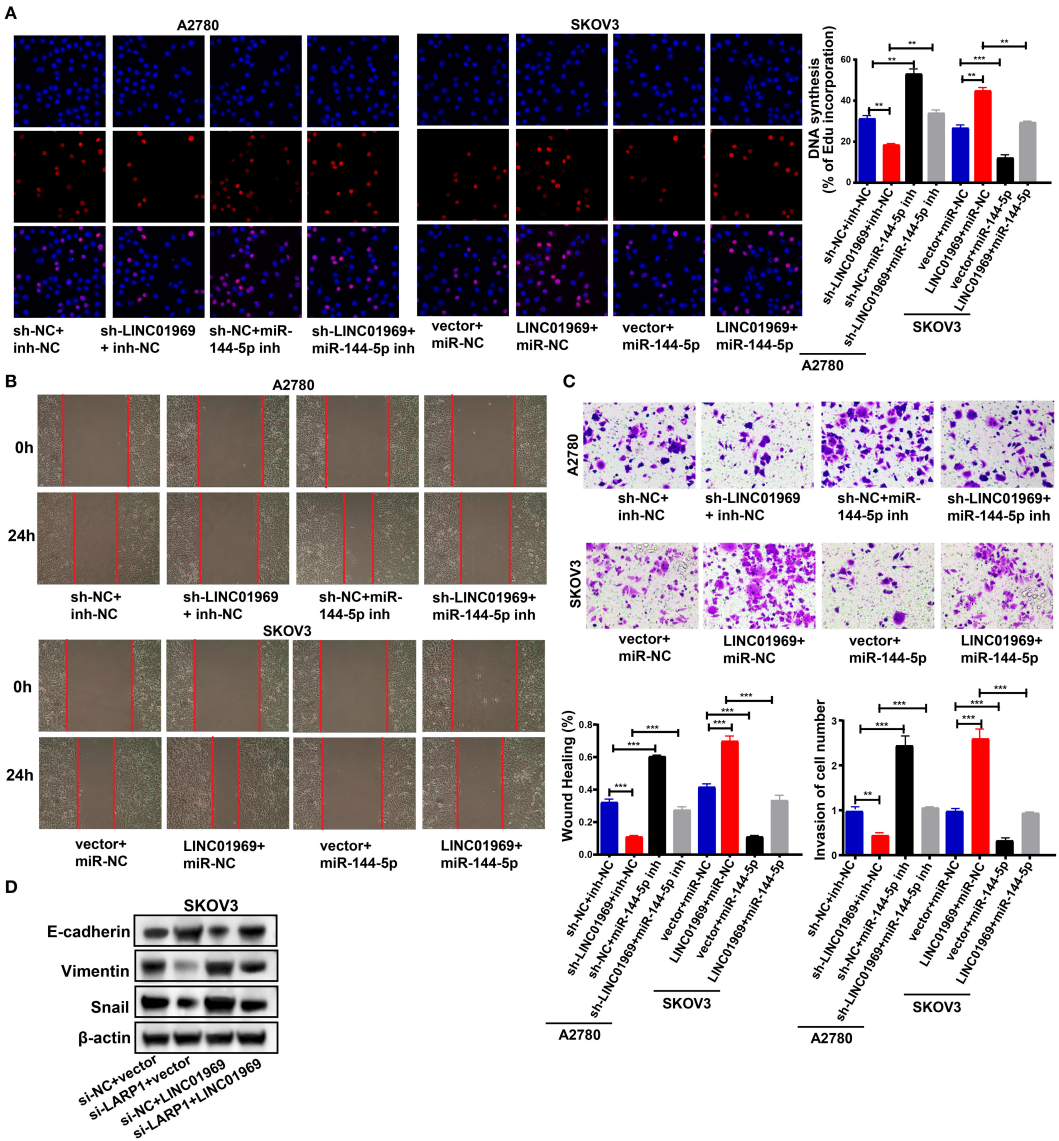
*LINC01969*/miR-144-5p axis regulates ovarian cancer (OC) progression. **(A)** Ethynyl-2-deoxyuridine (EdU) showed that miR-144-5p mimics treatment reversed the effect of *LINC01969* overexpression on OC cell proliferation. **(B)** Wound healing assay showed that miR-144-5p inhibitor treatment reversed the effect of *LINC01969* silencing on OC cell migration. **(C)** MiR-144-5p mimics treatment reversed the effect of *LINC01969* overexpression on OC cell invasion. **(D)** The expression of epithelial–mesenchymal transition (EMT) markers were determined using Western blot assay. Data were presented as represent the mean ± SD of three independent experiments; **P < 0.01, ***P < 0.001.

We then evaluated the effect of the *LINC01969*/miR-144-5p axis in OC. This was achieved by cotransfecting A2780 cells with the following combinations: sh-NC + inh-NC, sh-*LINC01969* + inh-NC, sh-NC + miR-144-5p inh, and sh-*LINC01969* + miR-144-5p inh. SKOV3 cells were cotransfected with the following combinations: vector + miR-NC, *LINC01969* + miR-NC, vector + miR-144-5p, and *LINC01969* + miR-144-5p. An EdU assay exhibited that transfection of sh-*LINC01969* inhibited cell proliferation, whereas miR-144-5p inhibitor promoted cell proliferation and reversed the effect of *LINC01969* silencing on A2780 cell proliferation (Figure 7A). Transfection of the miR-144-5p mimics reversed the effect of *LINC01969* overexpression on SKOV3 cell proliferation (Figure 7A). The results presented in Figures 7B,C show that cell migration and invasion were increased by *LINC01969* overexpression and miR-144-5p inhibitor but decreased by sh-*LINC01969* and miR-144-5p mimics. Conversely, transfection of miR-144-5p mimics reversed the cell invasion- and migration-promoting effects of *LINC01969* overexpression. Transfection of the miR-144-5p inhibitor reversed the effect of *LINC01969* silencing on cell migration and invasion. Next, we determined whether LINC01969 regulates EMT of OC cells through LARP1. EMT marker expression was evaluated using a Western blot assay. Knockdown of LARP1 increased E-cadherin expression and decreased Vimentin and Snail expression in OC cells (Figure 7D). In addition, knockdown of LARP1 reversed the EMT-promoting effect of LINC01969 overexpression (Figure 7D).

All these findings imply that *LINC01969* functions as a ceRNA for miR-144-5p to control *LARP1* expression and promote the proliferation, migration, and invasion of OC cells.

### *LINC01969* shRNA Blocks the Proliferation and Metastasis of OC Cells *in vivo*

**Figure 8:**
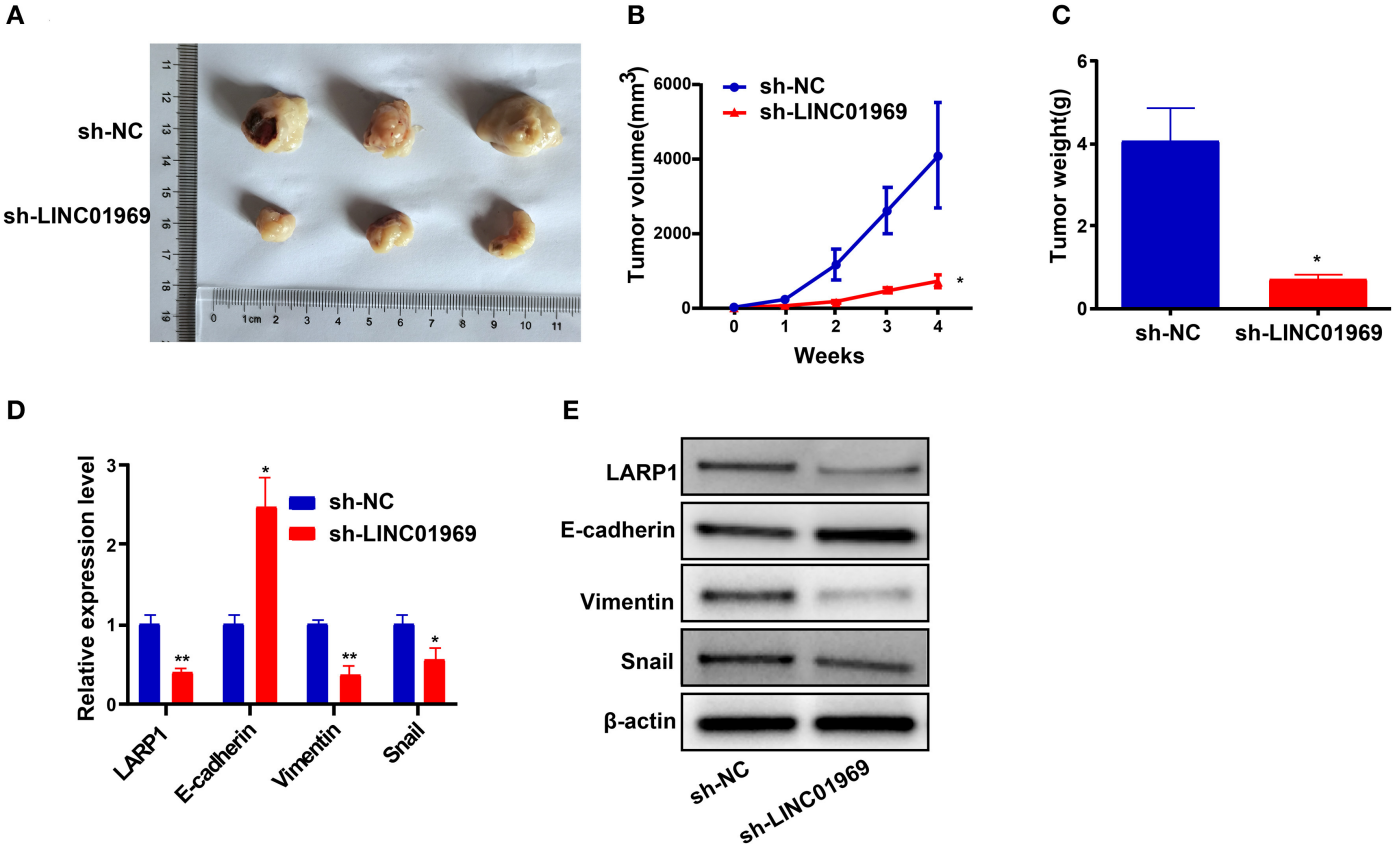
*LINC01969* short hairpin RNA (shRNA) curbs ovarian cancer (OC) cells to proliferate and metastasize *in vivo*. **(A)** Representative images of tumors from indicated orthotopic xenografts. **(B)** Tumor volume and **(C)** weight data of indicated orthotopic xenografts. **(D,E)** Messenger RNA (mRNA) and protein expression levels of *LARP1* and epithelial–mesenchymal transition (EMT)-related markers after *LINC01969* knockdown. Data were presented as mean ± SD; *P < 0.05, **P < 0.01.

We established xenograft models to confirm our *in vitro* findings *in vivo*. We injected *LINC01969*-silenced and non-silenced A2780 cells into nude mice. Then, tumor volume and weight were measured, and the tumors obtained from the mice are shown in Figure 8A. We found that *LINC01969* knockdown greatly slowed tumor growth (Figure 8B), and the *LINC01969* knockdown tumors were lighter at the end of the experiment (Figure 8C). qRT-PCR and Western blot assays showed that *LARP1*, Vimentin, and Snail expression levels were lower and E-cadherin expression levels were higher in mice with *LINC01969-*silenced tumors (Figures 8D,E), indicating that *LINC01969* knockdown decreased *LARP1* expression and OC cell EMT *in vivo*. These results show that *LINC01969* can promote OC growth and metastasis *in vivo*.

## Discussion

With the advancement of high-throughput sequencing technology, a growing number of lncRNAs have been identified. The roles of lncRNAs in diverse biological processes have been widely explored, and how lncRNAs regulate tumorigenesis has become a hot research topic. Mounting evidence has implicated various lncRNAs in the modulation of cancer cell proliferation, migration, and apoptosis (Jiang et al., 2018; Wu et al., 2018; Zhao et al., 2019; Chen et al., 2020). Likewise, we found a lncRNA, *LINC01969*, that was highly expressed in OC samples and was related to clinical stage and prognosis. The role of *LINC01969* in cancer has not yet been well-researched. Using tumor tissues from a population of OC patients (*n* = 41) and OC cell lines, we verified that *LINC01969* was indeed expressed at high levels in OC tissues and cell lines. Using functional assays, we revealed that *LINC01969* promoted the proliferation, migration, invasion, and EMT of OC cells. We then probed the action mechanism of *LINC01969* in OC.

LncRNA–miRNA–mRNA regulatory networks, in which lncRNAs act as ceRNAs for miRNAs to boost expression of specific mRNAs, are well-recognized (Feng et al., 2018; Yang et al., 2019). For example, lncRNA *FAM225A* facilitates nasopharyngeal carcinoma (NPC) tumor formation and metastasis by sponging miR-590-3p/miR-1275 as a ceRNA and increasing *ITGB3* expression (Zheng et al., 2019). In addition, the *LINC01287*/miR-298/*STAT3* feedback loop modulates the growth and EMT phenotype of hepatocellular carcinoma (HCC) cells (Mo et al., 2018). Knockdown of lncRNA *PVT1* reduces cell migration and invasion and increases cell apoptosis via miR-145-mediated inhibition of FSCN1 in esophageal carcinoma cell (Shen et al., 2019). Here, we identified MiR-144-5p as a target of *LINC01969* using starBase. MiR-144-5p was previously reported to function as an antitumor miRNA in renal cell carcinoma (Yamada et al., 2018) and OC progression (Song et al., 2018). Assays showed that miR-144-5p mimics inhibited the proliferation, invasion, and migration of SKOV3 cells. Moreover, upregulation of miR-144-5p partially reversed the effects of sh-*LINC01969* on the proliferation, invasion, and migration of OC cells.

*LARP1* was predicted as a target gene of miR-144-5p using starBase. *LARP1* encodes an RNA-binding protein that is necessary for cancer cell survival and ribosome biogenesis (Al-Ashtal et al., 2019). LARP1 functions as a molecular switch for mTORC1-mediated translation of an essential class of mRNAs (Hong et al., 2017) and posttranscriptionally regulates mTOR and contributes to cancer progression (Mura et al., 2015) by regulating cell division, apoptosis, and cell migration (Burrows et al., 2010). Hopkins TG et al. reported that *LARP1* promotes OC progression and enhances resistance to chemotherapy by differentially modulating the stability of diverse mRNAs (Hopkins et al., 2016). The results of the present research confirmed that *LARP1* is a downstream target gene of miR-144-5p and that *LINC01969* competitively bound to miR-144-5p to release *LARP1*, thereby modulating *LARP1* expression.

In conclusion, *LINC01969* acts as an oncogene and promotes the migration, proliferation, invasion, and EMT of OC cells through the miR-144-5p/*LARP1* axis. *LINC01969* might be a useful prognostic biomarker in OC.

## Data Availability Statement

The original contributions generated for the study are included in the article/Supplementary Material, further inquiries can be directed to the corresponding author.

## Ethics Statement

The studies involving human participants were reviewed and approved by the Ethics Committee of Liaoning Cancer Hospital & Institute. The patients/participants provided their written informed consent to participate in this study. The animal study was reviewed and approved by the Ethics Committee of Liaoning Cancer Hospital & Institute.

## Author Contributions

JZ made prosperous contributions to conception and design. JC and XL performed the experiments. JC wrote the draft manuscript. LY analyzed the data. XL made collection of data. All authors made contributions to the examination of the manuscript and approved the final manuscript for submission.

## Conflict of Interest

The authors declare that the research was conducted in the absence of any commercial or financial relationships that could be construed as a potential conflict of interest.
