# Supplementary material for: Long Non-coding RNA LINC01969 Promotes Ovarian Cancer by Regulating the miR-144-5p/LARP1 Axis as a Competing Endogenous RNA
Source: Front Cell Dev Biol. 2021 Feb 4;8:625730. doi: 10.3389/fcell.2020.625730 (PMC7889973; doi:10.3389/fcell.2020.625730)
Supplement: Supplementary file 1 [file Table_1.DOCX]

**Table S1.** **Sequences of primers for qRT-PCR related sequence**

| **Name** |  | | **Sequence** |
| --- | --- | --- | --- |
| LINC01969 | | Forward | 5’- CCCATCAGACTAGGAGCAGG -3’ |
|  |  | Reverse | 5’- GGACAGGACTGAAGAGGGAC -3’ |
| miR-144-5p | | Forward | 5′-CGGGCGATATCATCATATACTG -3′ |
|  |  | Reverse | 5′-GTGCAGGGTCCGAGGT -3′ |
| LARP1 | | Forward | 5'- AGGCTCCCATACCTAGCTGC -3' |
|  |  | Reverse | 5'- TGCCTTTGCGCTGTTTAGGAA -3' |
| E-cadherin | | Forward | 5’- GCTGGACCGAGAGAGTTTCC -3’ |
|  |  | Reverse | 5’- CAAAATCCAAGCCCGTGGTG -3’ |
| Vimentin | | Forward | 5'- CGGGAGAAATTGCAGGAGGA -3' |
|  |  | Reverse | 5'-AAGGTCAAGACGTGCCAGAG -3' |
| Snail | | Forward | 5'-TCGGAAGCCTAACTACAGCGA -3' |
|  |  | Reverse | 5'-AGATGAGCATTGGCAGCGAG -3' |
| GAPDH | | Forward  Reverse | 5′-CTCGCTTCGGCAGCACA -3′  5′-AACGCTTCACGAATTTGCGT -3′ |
